# Binding of doxorubicin to Sorcin impairs cell death and increases drug resistance in cancer cells

**DOI:** 10.1038/cddis.2017.342

**Published:** 2017-07-20

**Authors:** Ilaria Genovese, Annarita Fiorillo, Andrea Ilari, Silvia Masciarelli, Francesco Fazi, Gianni Colotti

**Affiliations:** 1 Department of Biochemical Sciences, Sapienza University, Rome, Italy; 2 IBPM-CNR Institute of Molecular Biology and Pathology, Italian National Research Council, Rome, Italy; 3 Department of Anatomical, Histological, Forensic & Orthopaedic Sciences, Section of Histology & Medical Embryology, Sapienza University, Rome, Italy

## Abstract

Sorcin is a calcium binding protein that plays an important role in multidrug resistance (MDR) in tumors, since its expression confers resistance to doxorubicin and to other chemotherapeutic drugs. In this study, we show that Sorcin is able to bind doxorubicin, vincristine, paclitaxel and cisplatin directly and with high affinity. The high affinity binding of doxorubicin to sorcin has been demonstrated with different techniques, that is, surface plasmon resonance, fluorescence titration and X-ray diffraction. Although the X-ray structure of sorcin in complex with doxorubicin has been solved at low resolution, it allows the identification of one of the two doxorubicin binding sites, placed at the interface between the EF5 loop the G helix and the EF4 loop. We show that Sorcin cellular localization changes upon doxorubicin treatment, an indication that the protein responds to doxorubicin and it presumably binds the drug also inside the cell, soon after drug entrance. We also demonstrate that Sorcin is able to limit the toxic effects of the chemotherapeutic agent in the cell. In addition, Sorcin silencing increases cell death upon treatment with doxorubicin, increases the accumulation of doxorubicin in cell nucleus, decreases the expression of MDR1 and doxorubicin efflux via MDR1.

The development of drug resistance is the leading cause of chemotherapy failure in cancer treatment. Elucidation of the mechanisms that confer simultaneous resistance to different drugs with different chemical structures and molecular targets – multidrug resistance (MDR) – has been a primary goal of cancer biologists during the past decades. Chemotherapy is the treatment of choice in metastatic cancer; by limiting drug’s effectiveness, MDR represents a major obstacle to this option.

Cancer cells can adopt several strategies to evade death induced by chemotherapeutic agents. These include changes in apoptotic pathways, increased DNA damage repair, drug inactivation, alteration of drug targets and increased expression of ABC transporters, able to pump xenobiotics (such as toxins or drugs) out of cells.^1^ Many cancer cells express large amounts of MDR1 (ABCB1, or P-glycoprotein 1), which confers them MDR.^2, 3, 4^

Sorcin (SOluble Resistance-related Calcium-binding proteIN) gene is located in the same chromosomal locus and amplicon as the ABC transporters MDR1 and MDR3, both in human and rodent genomes, and is highly conserved among mammals. Sorcin was initially labeled ‘resistance-related’, since it is co-amplified with MDR1 in multidrug-resistant cells.^5, 6^ While for years Sorcin overproduction was believed to be a by-product of the coamplification of its gene with P-glycoprotein genes,^7^ many recent reports have demonstrated that Sorcin plays a role in MDR, and pointed at a possible role as an oncoprotein.

Sorcin is one of the most highly expressed calcium-binding proteins in many tissues, and part of the 5% most expressed proteins of the human proteome (PaxDb). Importantly, Sorcin is overexpressed in many human tumors and MDR cancers.^8^ The level of Sorcin expression in leukemia patients inversely correlates with patients’ response to chemotherapies and overall prognosis. In parallel, Sorcin is highly expressed in chemoresistant cell lines and significantly upregulated in doxorubicin-induced MDR leukemia cell line K562/A02 over its parent cells. Sorcin overexpression by gene transfection increased drug resistance to a variety of chemotherapeutic agents in K562 cells, SGC7901 cells, ovarian and breast cancer. On the other hand, several studies have demonstrated that inhibition of Sorcin expression by RNA interference led to reversal of drug resistance in many cell lines.^8^

Recent data indicate that Sorcin participates in several processes that might contribute to MDR in human cancers, such as drug efflux regulation, apoptosis modulation and epithelial-to-mesenchymal transition (EMT) control.^8, 9^ Conflicting results are in literature on the effect of Sorcin overexpression and silencing on MDR1 expression and activity.^10, 11, 12, 13^ A complete understanding of the mechanisms and pathways by which Sorcin contributes to the MDR phenotype of tumor cells and an assessment of the overall diagnostic and therapeutic potential of sorcin in MDR are still missing.

Recently we have solved the crystal structure of apo- and calcium-bound human Sorcin, showing the mechanism of activation of the protein.^14^ Upon calcium binding Sorcin undergoes a large conformational change that exposes three pockets, hydrophobic surfaces involving the EF loop and EF5 hand (Pocket 1), EF2-EF3 (Pocket 2) and region EF1-EF3 (Pocket 3). This allows Sorcin to bind and regulate target proteins in a calcium-dependent fashion.^15, 16, 17, 18, 19, 20, 21^

Here we demonstrate that Sorcin binds doxorubicin directly and with high affinity and that it changes its cellular localization upon doxorubicin treatment and limits the toxic effects of doxorubicin in the cell; the low resolution structure of Sorcin in complex with doxorubicin allowed the identification of at least one chemotherapeutic drug binding site. We also demonstrate that Sorcin silencing increases cell death upon doxorubicin treatment, increases the accumulation of doxorubicin in cell nucleus, decreases the expression of MDR1 and doxorubicin efflux via MDR1.

## Results

### Sorcin binds doxorubicin and other chemotherapeutic drugs with high affinity

**Figure 1:**
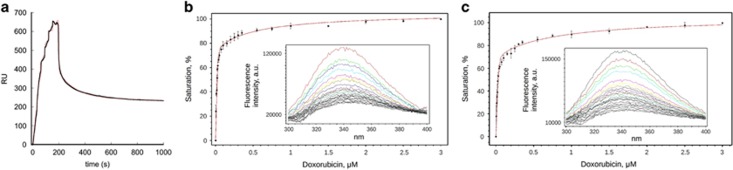
Sorcin binds doxorubicin with high affinity ***in vitro***. (**a**) SPR titration experiments in the presence of 500 *μ*M CaCl_2_ and (**b,c**) fluorescence titration experiments in the presence of 0.5 *μ*M EDTA: Doxorubicin binding to Sorcin (**b**) and SCBD (**c**) monitored by intrinsic fluorescence quenching. Each protein was incubated for 3 min at 25 °C in the presence of increasing concentration of ligand. The bars indicate the standard deviation for three independent experiments. The insets show the whole emission peak for each sample from one representative experiment. Both Sorcin and SCBD contain two binding sites for doxorubicin, with affinities in the nanomolar and low micromolar range

For surface plasmon resonance (SPR) experiments, two types of sensorgrams have been measured. OneStep-SPR experiments show that Sorcin is able to bind doxorubicin, paclitaxel and vinblastine, with high affinity, in the submicromolar range (Figure 1,Supplementary Figure S1); FastStep-SPR experiments (Figure 1a,Supplementary Figure S2) can be fitted with two binding sites, one in the nanomolar range and one in the low micromolar range (*K*_D1_=10 nM; *K*_D2_=1 *μ*M in the presence of calcium; *K*_D1_=22 nM; *K*_D2_=2 *μ*M in the presence of EDTA). Sorcin also binds cisplatin, with a *K*_D_=1.7 *μ*M (one binding site, Supplementary Figure S1). Fluorescence titrations (Figures 1b and c,Supplementary Figure S2) were carried out by measuring the fluorescence at 280 nm upon stepwise doxorubicin addition to Sorcin (Figure 1b) and to the Sorcin calcium-binding domain (SCBD, Figure 1c), comprising residues 32–198 of Sorcin. The fitting of fluorescence titrations for both Sorcin and SCBD are compatible with 2 doxorubicin binding sites (Figure 1, Supplementary Figure S2), with affinity constants in the same order of magnitude with respect to those measured by SPR experiments, that is, 1.4±1 and 734±396 nM for SCBD and 0.9±0.5 and 511±140 nM for Sorcin in the presence of EDTA (1.2 and 360 nM; 0.9 and 318 nM for Sorcin, in the presence of 1 and 5 mM magnesium, respectively): doxorubicin binding occurs at the C-terminal calcium-binding domain, since SCBD retains the binding sites. Signal shift was not detected, indicating that the environment of fluorophores did not change upon doxorubicin binding. The value obtained for *K*_D1_ is lower than protein concentration, condition that can cause an overestimation of the constant and large errors. We could not lower protein concentration due to the signal/noise ratio; however it can be assessed that *K*_D1_ is not greater than estimated.

Therefore, Sorcin, which was previously shown to increase resistance to a variety of chemotherapeutic agents, is able to bind directly and with high affinity doxorubicin and other chemotherapeutic drugs *in vitro*; this prompted further experiments to understand how such binding may contribute to increase drug resistance in cells as a function of its expression in the cell.

### Crystal structure of the Sorcin-doxorubicin complex

**Figure 2:**
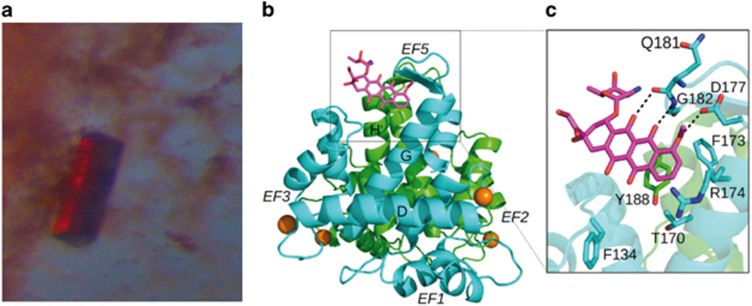
Sorcin calcium binding domain-doxorubicin complex. (**a**) Crystal and (**b**) crystal structure of Sorcin calcium binding domain-doxorubicin complex; (**c**) doxorubicin binding site at EF5 (pocket 1), stacked to Tyr188

Addition of 4 : 1 molar excess of doxorubicin to a clear, transparent solution of concentrated apo-Sorcin determines clouding of the solution, aggregation and precipitation of the protein (similar to the precipitation observed upon calcium addition), with formation of a red precipitate and the slow growth of red-colored crystals (Figure 2a).

Crystals of different intensity of red color grew depending on the amount of doxorubicin used for crystallization, ranging from 0.5 : 1 (colorless) to 2 : 1 (pink) to 15 : 1 (red) molar excesses. Emission spectra of the crystals grown from these solutions were recorded at 100 K in the Bessy facility, exciting at 473 nm. Changes in peaks intensity and a 25 nm red shift of the bands in high-amount doxorubicin (red crystals) were observed with respect to low-amount doxorubicin Sorcin crystals (Supplementary Figure S3). These changes are likely due to doxorubicin stacking to aromatic residues of the protein or doxorubicin dimerization, once bound.^22^

We solved the structure of the complex between SCBD and doxorubicin (doxo-SCBD) at quite low resolution (3.74 Å, PDB accession: 5MRA). The asymmetric unit contains two dimers (A–B and C–D). The structure contains 10 Mg^2+^ ions (3 bound to monomer A, 2 to monomer B, 3 to monomer C, 2 to monomer D). Doxorubicin is bound to the B monomer. The protein structure is similar to apo-Sorcin and apo-SCBD (PDB accessions: 4UPG, 1 GJY^14, 23^) (Figure 2b). The superimposition between the C*α* trace of doxo-SCBD with the C*α* trace of apo-Sorcin yields an rmsd of 1.11 Å, indicating that the structures are similar and therefore neither Mg^2+^ ions binding nor doxorubicin binding are able to promote the conformational changes induced by calcium ions in Sorcin. In accordance with binding experiments, doxorubicin binding occurs at two sites. Inspection of the Fo-Fc electronic density map allowed the identification of two peaks (Supplementary Figure S4): one close to the EF5 hand, which does not bind calcium in Sorcin and is responsible for dimer formation, and the other close to the D-helix connecting the EF2 and EF3 sites at the interface between the two dimers. We succeeded in modeling the doxorubicin molecule in the first site (close to the EF5) whereas it was not possible to model the doxorubicin molecule in the second site (close to the D helix) indicating both the low occupancy of the site and the flexibility of the doxorubicin molecule (Supplementary Figure S4). These sites have been previously identified as Pocket 1 and Pocket 2, able to bind protein targets, in another PEF protein, that is, PDCD6 (ALG-2).^24^

The binding of doxorubicin to SCBD in pocket 1 involves a stacking interaction of the drug with the aryl ring of Tyr188 of one monomer and interaction with Asp177, Gly182, Phe173 and Phe134 of the two-fold symmetry related monomer at the dimeric interface (Figures 2b and c). In the second putative site (pocket 2) doxorubicin likely interacts with Trp105 and Phe134. Probably doxorubicin binding to the second site would be facilitated by the binding of calcium ions which, as previously described,^14^ induce a conformational change promoting the movement of the D-helix and the exposure of hydrophobic interfaces.

In the structure, magnesium is bound to EF3 and to part of EF1 and EF2 sites, showing that in Sorcin the first three EF-hands can bind not only calcium, but also magnesium, with rather high affinity, and that EF3 is the site endowed with the highest affinity for divalent cations, responsible for Sorcin cation-dependent activation.

### Sorcin localization responds to doxorubicin treatment

**Figure 3:**
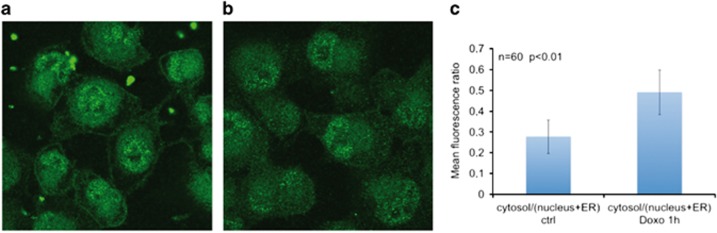
Sorcin localization changes upon doxorubicin treatment. Sorcin localization (green fluorescence) in (**a**) control H1299 cells and in (**b**) H1299 cells treated for 1 h with 0.6 *μ*M doxorubicin. (**c**) ratio between cytosol/(nucleus+ER) fluorescence (*n*=60 cells; *P*<0.01)

In H1299 lung cancer cell line, Sorcin (green fluorescence) localizes to cell membrane, nucleus, ER and cytosol, as already observed in other cellular systems.^17^ Upon treatment with doxorubicin, Sorcin localization changes with respect to control: after 1-h doxorubicin treatment, cytosolic Sorcin localization increases and nuclear, ER and membrane localization decreases; the ratio of cytosol/(nuclear+ER) Sorcin fluorescence increases by 77% (from 0.278 to 0.491, number of cells=60, *P*<0.01, Figure 3). This is a clear indication that Sorcin localization responds to doxorubicin treatment and that Sorcin presumably binds doxorubicin also in the cell, upon drug entry.

### Effect of Sorcin expression on doxorubicin uptake and toxicity, and cell death

**Figure 4:**
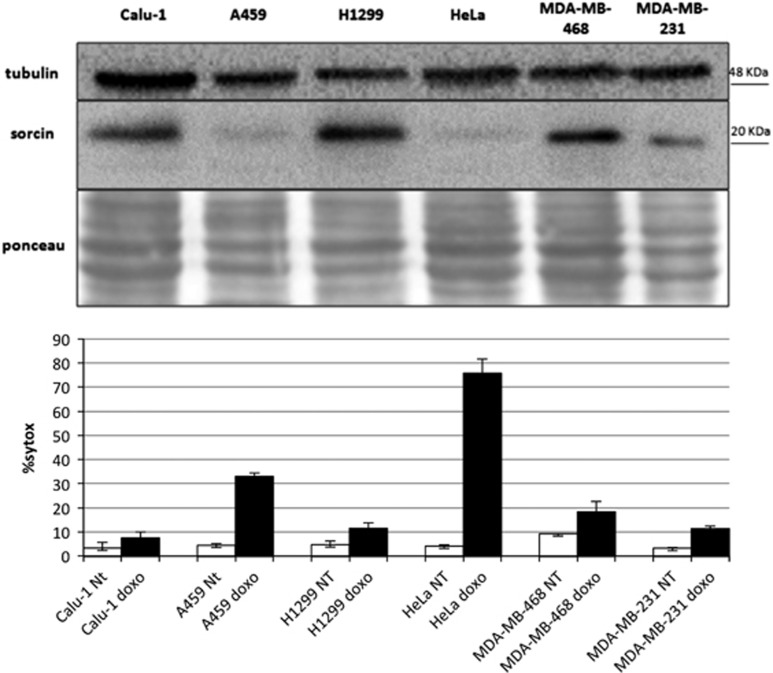
Sorcin expression *versus* cell death. (top) Western blot experiment showing the expression of Sorcin in lung carcinoma Calu-1, A459 and H1299 cells; cervix adenocarcinoma HeLa; breast adenocarcinoma MDA-MB-468 and MDA-MB-231. (bottom) Cell death is increased upon 24 h doxorubicin (0.6 *μ*M) treatment in A549 and HeLa cells, where Sorcin expression level is reduced by >80% with respect to H1299 cells

Sorcin is expressed at high levels in human and in many cell lines (PaxDB). We have analyzed Sorcin expression in different cell lines from lung, cervix and breast cancers and we evidenced that Sorcin is expressed in all tested cell lines, but the levels differ even by more than one order of magnitude between different lines. In particular, Sorcin is highly expressed in lung cancer cell lines Calu-1 and H1299, that we have selected for further studies, and in breast cancer cell lines MDA-MB-231 and MDA-MB-468, while low Sorcin expression levels were observed in lung A549 and in cervical cancer HeLa cells (Figure 4).

Sorcin high level of expression occurs in cell lines rather resistant to cell death upon treatment with doxorubicin, as H1299, Calu-1 and MDA-MB-468 cells, while A459 and HeLa cell lines, where Sorcin expression is lower by about 90%, are more sensitive to doxorubicin treatment (Figure 4). To support the relevance of Sorcin in doxorubicin treatment response, we proceeded with Sorcin silencing experiments. In all tested cell lines, siRNA cds3 effectively silences Sorcin expression, by at least 85% after 24–48 h (Supplementary Figure S5A). In the H1299 line, 94±3% silencing occurs. Interestingly the silencing of Sorcin expression is also maintained upon doxorubicin treatment (Supplementary Figure S5B).

**Figure 5:**
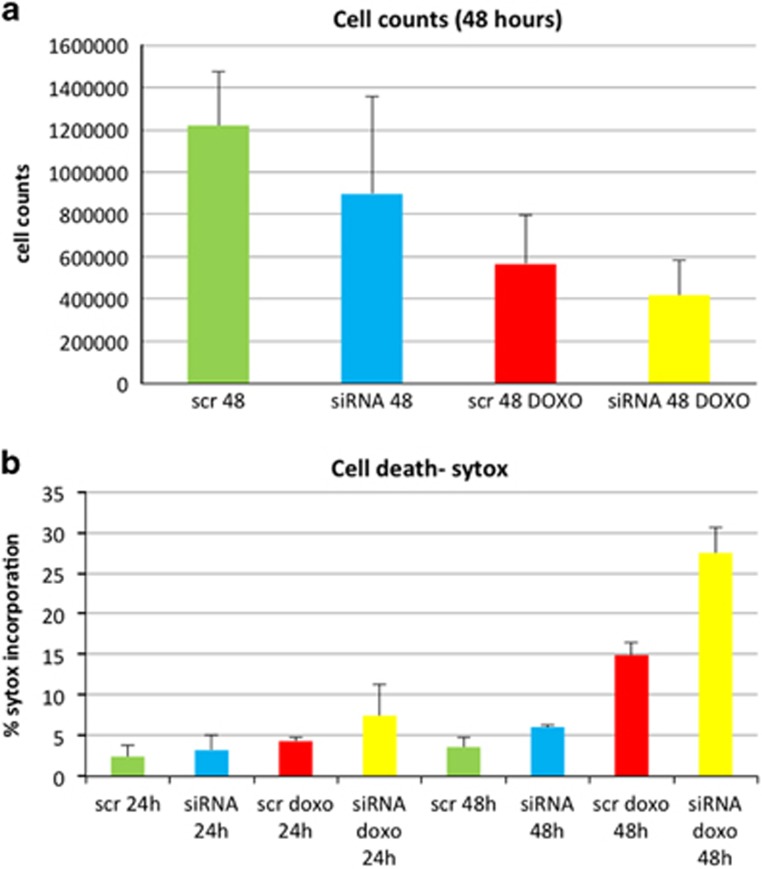
Sorcin silencing increases cell death upon treatment of H1299 cells with 0.6 *μ*M doxorubicin. (**a**) Cell count and (**b**) cell death percentage upon treatment of H1299 cells with scrambled siRNA or Sorcin siRNA in control and doxorubicin-treated cells

Sorcin silencing by siRNA cds3 (*versus* control experiments with scrambled siRNA) slightly increases cell death (Figures 5a and b,Supplementary Figure S6), as shown by both cell count and by flow cytometry experiments on cells stained with Sytox blue, a cyanine dye that is completely excluded from live eukaryotic cells.

Upon Sorcin silencing, doxorubicin-dependent cell death is markedly increased in H1299 cells (Figure 5): upon treatment with scrambled siRNA and 0.6 *μ*M doxorubicin, the percentage of dead H1299 cells increases from 3.4% (control) to 4.6% and 16.3% (24 and 48 h after doxorubicin treatment, respectively), while upon treatment with Sorcin-directed siRNA and 0.6 *μ*M doxorubicin, the percentage of dead H1299 cells increases from 4.5% (control) to 10.3% and 29.7% (+124% and +82%, 24 and 48 h after doxorubicin treatment, respectively).

**Figure 6:**
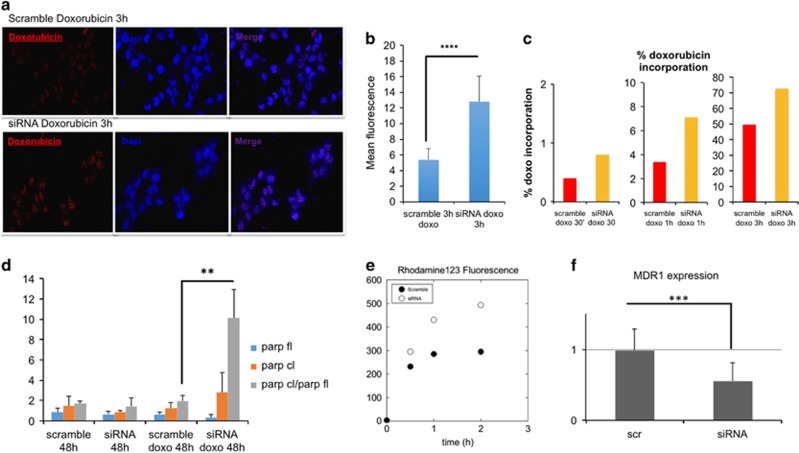
Sorcin silencing increases doxorubicin accumulation in H1299 cells. (**a**) confocal microscopy images, showing the nuclear accumulation of doxorubicin upon 3 h treatment in H1299 cells treated with scrambled siRNA (top) or with Sorcin siRNA (bottom); (**b**) nuclear doxorubicin incorporation by fluorescence quantification (*n*=60 cells; *P*<0.0001); (**c**) time-dependent quantification of doxorubicin incorporation in H1299 cells by FACS; (**d**) upon 48 h doxorubicin treatment, in Sorcin-silenced cells PARP cleavage is increased with respect to control cells (*n*=3, *P*<0.01). Sorcin silencing decreases MDR1 activity and expression with respect to control cells: (**e**) Rhodamine123 fluorescence is increased (and therefore its efflux is decreased) and (**f**) MDR1 expression is decreased in Sorcin-silenced H1299 cells with respect to control cells (*n*=3, *P*<0.001)

Further, Sorcin silencing increases doxorubicin entry in H1299 cell nuclei by 140% as shown by analysis of confocal microscopy experiments (Figures 6a and b). FACS experiments (Figure 6c,Supplementary Figure S7) show that upon treatment with scrambled siRNA and 0.6 *μ*M doxorubicin, the percentage of doxorubicin incorporation increases from 0.4% after 30 min to 3.4% after 1 h to 49.6% after 3 h doxorubicin treatments, while upon treatment with Sorcin-directed siRNA and 0.6 *μ*M doxorubicin, the percentage of doxorubicin incorporation increases from 0.8% after 30 min to 7.1% after 1 h to 72.7% after 3 h doxorubicin treatments (+100%, +109%, +47%, respectively). After 5 h incubation with doxorubicin, the buffering capacity of Sorcin is almost lost (Supplementary Figure S7).

In the presence of high levels of Sorcin, doxorubicin is therefore prevented from entering the nuclei of H1299 cells, and the cells are protected from drug-dependent DNA damages.

Sorcin protects cells, while Sorcin silencing increases doxorubicin-dependent Poly(ADP-ribose)polymerase (PARP) cleavage (Figure 6d,Supplementary Figure S8), an apoptotic marker. In cells treated with Sorcin-directed siRNA, 48 h after treatment with 0.6 *μ*M doxorubicin, the levels of cleaved PARP are higher than in control cells. An even higher increase of doxorubicin-dependent PARP cleavage upon Sorcin silencing in doxorubicin-treated cells can be measured by calculating the ratio between the intensities of cleaved *versus* full-length PARP.

### Effect of Sorcin expression on MDR1 expression and activity

The effect of Sorcin expression on doxorubicin uptake and toxicity can be explained in part by the direct binding of doxorubicin by Sorcin, that may prevent the drug entry in the nucleus. However, doxorubicin is also a substrate of the efflux pump MDR1,^2^ whose gene is located in the same amplicon of Sorcin gene.^5^ Conflicting results are in literature on the effect of Sorcin expression on MDR1 expression and activity.^10, 11, 12, 13^

Figures 6e and f show that Sorcin silencing decreases both MDR1 expression and activity in H1299 cells, as already demonstrated in A549 cells^25^ by about 40%. In cells treated with Sorcin-directed siRNA the MDR1-mediated efflux of rhodamine123 is substantially decreased with respect to control cells treated with scrambled siRNA, showing a decrease of the activity of MDR1 in Sorcin-silenced cells (Figure 6e,Supplementary Figure S9): the level of intracellular rhodamine123 in H1299 cells treated with Sorcin-directed siRNA is increased by 27, 51 and 67% (upon 30 min, 1 h and 2 h incubation with the MDR1 substrate, respectively) with respect to control cells.

MDR1 expression level is also strongly decreased by Sorcin silencing: in cells treated with Sorcin-directed siRNA, a 45% decrease in MDR1 level occurs with respect to H1299 control cells (Figure 6f, Supplementary Figure S10).

## Discussion

The high level of expression of Sorcin in many tumors, especially the MDR ones, the inverse correlation of Sorcin expression with patients’ response to chemotherapies and overall prognosis, and reversal of drug resistance upon Sorcin expression by RNA interference have recently struck the attention of many scientists. In particular, Sorcin has been shown to be upregulated in doxorubicin-induced MDR K562/A02 leukemia cells over their parent cells, and Sorcin overexpression has been demonstrated to increase drug resistance to doxorubicin, etoposide, homoharringtonine, vincristine, taxol, cisplatin and 5-fluorouracil in several cancer cells.^6, 26, 27, 28, 29, 30, 31, 32^ Further, many studies have demonstrated Sorcin participation in several processes, such as drug efflux regulation, apoptosis modulation and EMT control, that might contribute to the onset of MDR in human cancers.^8, 9^

However, the molecular basis of Sorcin-linked processes that determine MDR has not been elucidated yet.

Here, we show for the first time that Sorcin is itself able to bind directly and with high affinity chemotherapeutic agents able to induce Sorcin-dependent MDR, such as cisplatin, vinblastine, paclitaxel and in particular doxorubicin. SPR and fluorescence titration experiments demonstrated that Sorcin binds doxorubicin with high affinity and that there are at least two doxorubicin-binding sites. The low resolution X-ray structure of the complex allowed the identification of at least one of these sites. Doxorubicin binds to regions that in PDCD6, structurally similar to Sorcin, are involved in ALIX-Abs peptide binding:^24^ binding occurs with 100% occupancy and high affinity to Pocket 1 and with lower occupancy to Pocket 2. Sorcin can therefore use the same pockets in different fashions: they not only can bind calcium channels and other proteins (RyR receptors, SERCA, NCX1), and regulate their activity, thereby increasing Ca^2+^ accumulation in the endoplasmic (ER)/sarcoplasmic reticulum (SR), and increasing resistance to ER stress,^17, 19, 20, 21, 33, 34^ but can also bind chemotherapeutic drugs with high affinity, decreasing doxorubicin-dependent toxicity and cell death. In addition, the present study shows that in H1299 cells expressing high Sorcin levels, upon doxorubicin treatment Sorcin acquires a more diffused cytosolic pattern, implying that doxorubicin can be bound and sequestered by Sorcin (which is one of the most expressed calcium-binding proteins) in the cytosol, before it can translocate to the nucleus and exert its toxic effects at cellular level.

These experiments contribute to elucidate the mechanisms of drug resistance to chemotherapeutic agents in highly Sorcin-expressing cells. Doxorubicin binds at the D-helix connecting EF2 and EF3 sites of Sorcin, thereby covering this area and impairing the Trp105-based interaction with its targets located on cell membranes and ER surface^14, 16, 21, 35, 36^ and at Tyr188 and Arg174 residues, belonging to the putative Nuclear Localization Sequence of Sorcin,^17^ thereby hampering Sorcin translocation to nucleus.

Doxorubicin sequestration impairs its chemotherapeutic action, based on drug entry in the nucleus and its intercalation in the DNA, and allows its MDR1-based extrusion. Possibly, in a first phase, MDR may depend predominantly on doxorubicin sequestration. In the longer term, sequestration reaches saturation, and MDR depends predominantly on the higher MDR1-based extrusion.

We also show that when Sorcin expression is decreased, the cells become sensitive to doxorubicin: the chemotherapeutic drug can accumulate in the nucleus, where it exerts cytotoxic effects by inhibiting topoisomerase II, thereby generating free radicals and DNA damages, and activating death pathways via activation of caspases, disruption of mitochondrial membrane potential or mitotic catastrophe accompanied by senescence-like phenotype.^37^ Chemotherapeutics binding to Sorcin is a fast process that results in fast cellular response to drug administration: Sorcin, one of the most highly expressed calcium-binding proteins, can act as a buffer for drugs, within a limited time span. Sorcin is a signaling protein, because of its ability to respond rapidly to calcium binding and, as we have demonstrated, to other molecules, such as doxorubicin. In addition, alteration of the cellular levels of Sorcin is a slower process, that impacts on MDR1 expression and that results in another mechanism of Sorcin-dependent drug resistance: Sorcin overexpression induces MDR1 expression via a cAMP-response element (CRE) of the MDR1 gene, and therefore through activation of the CREB pathway, by increasing CREB1 phosphorylation and the binding of CREB1 to the CRE sequence of mdr1 promoter.^13^ Sorcin silencing determines a decrease of activity and expression of MDR1, that pumps doxorubicin and many other drugs outside of the cell, in line with data from other resistant lung carcinoma cells or other tumors.^13, 25^ Sorcin silencing, combined to doxorubicin treatment, make cells prone to death. Sorcin also participates in other mechanisms related to oncogenesis and MDR onset, since it increases escape from apoptosis, by preventing ER stress and the unfolded protein response, by upregulating Bcl-2 and decreasing Bax expression.^17, 33, 38, 39, 40^

The structure of doxorubicin-bound Sorcin deserves some comments. Doxorubicin binding increases the disorder of the crystal and decreases the resolution of the structure with respect to those from unliganded protein. Addition of doxorubicin to a solution of concentrated apo-Sorcin determines clouding of the solution, aggregation and precipitation of the protein, followed by formation of a red precipitate and the slow growth of red-colored crystals. However, the structure of the Sorcin-doxorubicin complex is very similar to that of the apo protein, possibly because of the presence of magnesium in the crystallization solution. Magnesium binds to EF1, EF2 and EF3, in line with the affinity for calcium, which follows the order EF3>EF2>EF1.^36^ While calcium binding to Sorcin determines a large conformational change and protein activation, neither magnesium binding nor doxorubicin binding alters the structure. This can be due to the smaller ionic radius of magnesium with respect to calcium, and to crystal lattice forces that may favor an apo-like conformation. Generally speaking, calcium-dependent regulation of cellular activities is based on transient and/or local increase of Ca^2+^ concentration from 100 nM to low micromolar, while Mg^2+^ concentration remains constant at about 0.5–2 mM, that is, 2–4 orders of magnitude higher than Ca^2+^. Usually EF-hand proteins discriminate against Mg^2+^, being evolved to take advantage of the larger ionic radius and the less stringent demands on coordination ligands of Ca^2+^.^41^ Sorcin, at least in conditions where Mg^2+^ is very concentrated, is able to bind with full occupancy this ion. However, possibly calcium binding can elicit conformational changes that may lead to a better exposure of doxorubicin-binding pockets.

Overall our study demonstrates that Sorcin is able to bind directly and with high affinity doxorubicin and other chemotherapeutic drugs, and that this contributes to the generation of the MDR phenotype. This work, together with other recent papers, shows that Sorcin can be a useful marker of MDR and may represent a therapeutic target for reversing tumor MDR.

## Materials and methods

### Surface plasmon resonance (SPR) experiments

SPR experiments were performed with a SensiQ Pioneer apparatus. Wild-type human Sorcin was immobilized via amine coupling onto a COOH5 sensorchip, previously chemically activated by 100 *μ*l injection of a 1 : 1 mixture of *N*-ethyl-*N*′-3-(diethylaminopropyl)carbodiimide (200 mM) and *N*-hydroxysuccinimide (50 mM). Immobilizations were carried out in 20 mM sodium acetate at pH 4.5; the remaining ester groups were blocked by injecting 100 *μ*l of 1 M ethanolamine hydrochloride at pH 9.5.

The amount of immobilized Sorcin was detected by mass concentration-dependent changes in the refractive index on the sensorchip surface, and corresponded to about 5000 resonance units (RU).

Samples of analytes (doxorubicin, cisplatin, vinblastine and paclitaxel) were dissolved in 100% DMSO at a concentration of 10 mM, and subsequently diluted in sterile HEPES 20 mM, pH 7.4, NaCl 150 mM, 500 *μ*M CaCl_2_ (or EDTA) 0.005% surfactant P-20 to yield 2% DMSO final concentration (HSP-2%D buffer) and final drug concentration: 200 *μ*M. Further dilutions and all the experiments were carried out at 25 °C in degassed HSP-2%D buffer.

For FastStep experiments, the analytes were automatically diluted in HSP-2%D and injected by seven serial doubling steps (step contact time=30 s, nominal flow rate=100 *μ*l/min). At the following time points: (1) 0–30 s; (2) 31–60 s; (3) 61–90 s; (4) 91–120 s; (5) 121–150 s; (6) 151–180 s; (7) 181–198 s, analyte concentrations were: (1) 1.25 *μ*M; (2) 2.5 *μ*M; (3) 5 *μ*M; (4) 10 *μ*M; (5) 20 *μ*M; (6) 40 *μ*M; (7) 80 *μ*M. For OneStep experiments, Taylor dispersions were exploited to generate analyte concentration gradients that provide high-resolution dose response in single injections. Full analyte titrations were recorded in HSPC-2%D over four orders of magnitude in concentration, up to 80 *μ*M.

In both FastStep and OneStep experiments, the increase in RU relative to baseline indicates complex formation between the immobilized Sorcin ligand and the analytes. The plateau region represents the steady-state phase of the interaction. The decrease in RU after 198 s in FastStep experiments, or after 350 s in OneStep experiments, indicates analyte dissociation from the immobilized Sorcin after HSP-2%D buffer injection. As a negative control, sensor chips were treated as described above in the absence of immobilized Sorcin. Values of the plateau signal at steady-state (Req) and full fittings with 1, 2 and 3 sites were calculated from kinetic evaluation of the sensorgrams using the Qdat 4.0 program.

### Fluorescence titrations

Static fluorescence measurements were performed at 25 °C with a Horiba Fluoromax-4 spectrofluorometer using 1-cm path-length quartz cuvettes (slit width: 5 nm in excitation and emission). Fluorescence measurements were performed on Sorcin and SCBD, a shorter construct missing the first 32 residues, at two different concentrations: 30 nM and 37 nM, in Tris-HCl 10 mM, pH 7.5 and EDTA (0.5 *μ*M) or MgCl_2_ (1 mM or 5 mM). The excitation wavelength was set at 280 nm and emission spectra were collected in the 300–400 nm range. Triplicate samples were measured; each figure represents the average of three experiments.

Maximum emission occurs at 340 nm for SCBD and 338 nm for Sorcin. Upon doxorubicin addition, fluorescence quenching was observed to a maximum extent of about 60% in saturating condition. For each sample fluorescence was measured after 3 min of incubation.

Since doxorubicin absorbs light at 280 nm, fluorescence measurements are affected by the inner-filter effect. The following formula was employed for correction: Fcor=Fobs10^[(Aex)/2], where Fcor and Fobs are the corrected and observed fluorescence intensities, respectively, whereas Aex is the absorbance of each concentration of ligand at 280 nm [IFE-correction]. The effect is negligible at the concentrations of doxorubicin used (5–3000 nM). Data were fitted with the software Qtiplot assuming two independent binding sites. The equation used for data fitting is the weighted sum of two independent binging events: *K*((*k*+*c*+*x*)−sqrt((*k*+*c*+*x*)^2–4*cx*^))/(2*c*)+*H*((*h*+*c*+*x*)−sqrt((*h*+*c*+*x*)^2–4*cx*^))/(2*c*), where *c* is protein concentration, *k* and *h* are the two binding constants, *K* and *H* are the fraction of signal due to each binding event.

### Crystallization, data collection and structure solution

Crystallization experiments were performed with both human Sorcin and SCBD. Automated crystallization screening and by-hand optimization were carried out at 298 K by vapor diffusion method.

At first soaking technique was attempted but, while doxorubicin is deep red, the crystals stayed uncolored; then we moved to co-crystallization. Since Sorcin precipitates in presence of doxorubicin excess, trials were set up by adding the ligand to the crystallization drop (0.4 *μ*l of 0.5 mM protein+0.4 *μ*l of reservoir+0.1 *μ*l of 30 mM doxorubicin) to a ligand/protein ratio of about 15. Colored crystals, from light pink to red, grew in many conditions but most of them resulted in poor diffraction. The best data set collected was at 3.7 Å resolution, from a SCBD crystal grew in 0.2 M MgCl_2_, 0.1 M Tris-HCl pH 7, 2.5 M NaCl. The crystals were cryoprotected by adding 40% w/v glucose to the mother liquor.

A single wavelength (0.9677 *λ*) data set was collected at ESRF at 100 K on the ID30-A3 MASSIF3 beamline equipped with a Eiger-X-4M detector and processed with XDS.^42^ Crystal parameters and data collection statistics are reported in Table 1.

The structure was determined by molecular replacement with the program MOLREP^43^ (CCP4 suite) using the structure of the calcium-free human Sorcin (PDB entry 4UPG) as search model. Refinement was performed using the maximum-likelihood method with the program REFMAC^44^ and model building with the program Coot.^45^

Fluorescence emission spectra of SCBD-doxorubicin crystals were collected at ESRF ID29S at 100 K and excitation wavelength 473 nm. The experimental setup is described in more detail in a paper by Royant *et al.*^46^

### Cell cultures and western blots

H1299, Calu-1, A459 human lung carcinoma, HeLa human cervix adenocarcinoma, MDA-MB-468 and MDA-MB-231 breast adenocarcinoma cell lines were cultured in DMEM medium (Gibco, Invitrogen, Thermo Fisher Scientific, Waltham, MA, USA) with 10% FBS (v/v) and 5% Penicillin/Streptomycin (v/v) at 37 °C in a balanced air humidified incubator with 5% CO_2_.

The cells were lysed in a 2% SDS lysis buffer (25 mM Tris-HCl at pH 7.5, 100 mM NaCl, 3 mM EDTA, 7% glycerol) with: NaF 1000 ×, NaVO_3_ 100 ×, Na_4_PO_7_ 20 ×, Aprotinin 1000 ×, Leupeptin 1000 ×, PMSF 100 × protease and phosphatase inhibitors as final concentrations.

Extracts were sonicated for 10 s and centrifuged at 12 000 r.p.m. for 10 min to remove cell debris. Lysates were quantified in proteins content with Pierce BCA protein assay kit (Thermo Fisher Scientific, Waltham, MA, USA) according to the manufacturer’s instructions.

Thirteen percent acrylamide-bisacrylamide SDS gel electrophoreses were run for sorcin, and 7% SDS-PAGE were run for PARP and MDR1. Proteins lysate content was checked by S-Ponceau staining. Western blotting analysis was performed with the following antibodies: rabbit polyclonal anti-human sorcin (home-made^14^), mouse monoclonal anti-PARP (Cell Signalling, Danvers, MA, USA, #9532), mouse monoclonal anti-MDR1 (Santa Cruz Biotechnology, Heidelberg, Germany, sc-13131), mouse monoclonal anti-tubulin (Sigma-Aldrich, Darmstadt, Germany, cat. T5168) and mouse monoclonal anti-βactin (Santa Cruz Biotechnology, sc-81178). Goat secondary anti-mouse and anti-rabbit antibodies conjugated to horseradish peroxidase were used (Bio-Rad, Hercules, CA, USA, cat. 170-6515, 170-6516). Immunostained bands were detected by chemiluminescence (Chemidoc, Bio-Rad).

### Doxorubicin treatment and silencing for sorcin

We performed a dose-response curve (0.1 *μ*M, 0.3 *μ*M, 0.6 *μ*M, 1.0 *μ*M); 0.6 *μ*M is the dose resulting in the best evaluation of time-dependent accumulation, and is compatible with doxorubicin plasma concentration 15 min to 2 h after treatment of many different types of cancer.^47^ Doxorubicin concentration (0.6 *μ*M) was used for most experiments. H1299 cells were transfected with a solution composed by Optimem medium (Gibco, Invitrogen, Thermo Fisher Scientific), Lipofectamine RNAimax (Invitrogen, Thermo Fisher Scientific, Waltham, MA, USA, cat.13778-030) and a final concentration of 500 pM siRNA for sorcin (CDS3 and 3′UTR) (IDT sequence to CDS3-exon3: 5′-GAUAGAUGCUGAUGAAUUGCAGAGA-3′ sequence to 3′UTR-exon8: 5′-AGCUGUACACUUUCAAGUAAGAUCT)-3′, according to the manufacturer’s instructions. CDS3 siRNA silences both sorcin isoforms.^18^ After 48 h of transfection, the medium was replaced with fresh DMEM (Gibco, Invitrogen, Thermo Fisher Scientific) containing 0.6 *μ*M doxorubicin. To evaluate doxorubicin incorporation, cells were treated with the drug in time-course experiments (30 min to 3 h incubation for cytofluorimetry, 3 h and 5 h for confocal microscopy). The analysis of biological effects of sorcin silencing was performed 24 h and 48 h after treatment.

### Doxorubicin uptake (confocal microscopy and FACS)

The uptake of doxorubicin was evaluated through confocal microscopy and FACS (Fluorescent-activated Cell Sorting) thanks to the autofluorescence of the molecule (excitation wavelength 470 nm; emission wavelength 585 nm). To avoid cells drug saturation the analysis was performed between 30 min and 5 h incubation.

For confocal microscopy, the medium was removed from the H1299 cells, then washed with PBS. The cells were fixed in 2% paraformaldehyde for 10 min, washed in PBS and incubated 7 min in TO-PRO-3 (Invitrogen, Thermo Fisher Scientific, Waltham, MA, USA, cat. T3605), dilution 1:3000. To avoid fluorophore quenching, samples were covered with Vectashield Mounting Medium (Vector Laboratories, Burlingame, CA, USA, cat. H-1000). Confocal images of slides were acquired at a Leica laser scanning microscope TCS-SP2.

In order to have a quantitative readout on doxorubicin incorporation we performed flow cytometry with CyAn ADP and Summit 4.3 software. The cells were dislodged with trypsin 0.05% (Gibco, Invitrogen, Thermo Fisher Scientific), the emission of doxorubicin was evaluated at 573 nm, and cells were gated as shown in supplementary results. Data were analyzed with FCS4 express software.

### Sytox blue assay and cell counts

To evaluate cell death we performed assays with Sytox Blue Dead Cell Stain, for flow cytometry (Molecular Probes, Invitrogen, Thermo Fisher Scientific, Waltham, MA, USA, cat. MP34857). According to the manufacturer’s instructions, 200 000 cells were sampled for each condition and incubated 15 min at room temperature with Sytox blue 1:1000 dilution. The samples were acquired at CyAn ADP by using Summit 4.3 software. The fluorescence excitation of nucleic acids of dead cells was measured with 405 nm violet laser light. Data were analyzed with FCS4 express software.

Lung, breast cancer cell lines and HeLa cells were treated 48 h with 0.6 *μ*M doxorubicin and the rate of Sytox blue incorporation was evaluated as aforementioned.

Although this assay is very reliable, we evaluated the effect of sorcin silencing on cell death with the traditional method of cell counts. The cells were dislodged diluted 1:1 with Trypan blue dye and counted in triplicates in a burker cell counting chamber.

### Sorcin localization, confocal microscopy

H1299 cells were treated 1 h with 0.6 *μ*M of doxorubicin and processed as aforementioned for confocal microscopy purpose. After paraformaldehyde fixation, cells were incubated 30 min with a 1:200 dilution of primary antibody against Sorcin and, after PBS 1 × washing steps, 30 min with Alexa Fluor 488 (Molecular Probes, Invitrogen, Thermo Fisher Scientific)-conjugated secondary antibody against rabbit was used at a 1:500 dilution. A Leica laser scanning microscope TCS-SP2 device was used and images were acquired with Leica confocal software.

### Rhodamine123 incorporation

To ascertain whether sorcin silencing affects MDR1 functionality in pumping out the drugs from the cell, we performed a rhodamine123 accumulation assay. This dye is extruded outside the cells by MDR1/MDR4 pumps. First the cells were silenced for 48 h, as mentioned, then a time course accumulation assay was performed. We considered 250 000 cells each time point (30 min, 1 h, 2 h) and the assay was carried out incubating the samples at 37 °C in RPMI 1640 medium without and with 1 *μ*M rhodamine123.

After incubation the samples were pelleted and washed twice in ice cold PBS 1 ×. Then they were analyzed at CyAn ADP by using Summit 4.3 software. The results were evaluated with FCS4 express software.

## Figures and Tables

**Table 1:** Crystal parameters, data collection statistics and refinement statistics

PDB code	5MRA
Space group	P2_1_ 2_1_ 2_1_
Cell parameters (Å)	a=92.36, b=104.98, c=113.42
Asymmetric unit, residues	Tetramer, 166 per monomer
N° of bound ions	10 Mg^++^
	
*Data reduction*	
Resolution range (Å)	48.31–3.74 (3.96–3.74)
Unique reflections	11800 (1845)
Completeness (%)	99.0% (97.2%)
Redundancy	6.53 (6.47)
*R*_merge_ (%)	10.2 (113.3)
CC(1/2)	99.9 (64.7)
*I/σ*(*I*)	11.81 (1.45)
	
*Data refinement*	
Resolution range (Å)	48.31–3.74 (3.83–3.74)
*R*_cryst_ (%)	19.6 (35)
*R*_free_ (%)	28.5 (37)
rms (angles) (°)	1.324
rms (bonds) (Å)	0.01
Wilson B-factor (Å^2^)	162.2
Residues in core regions of the Ramachandran plot (%)	90
Residues in allowed regions of the Ramachandran plot (%)	10

Values in parentheses are for the highest-resolution shell
