# Supplementary material for: Binding of doxorubicin to Sorcin impairs cell death and increases drug resistance in cancer cells
Source: Cell Death Dis. 2017 Jul 20;8(7):e2950–. doi: 10.1038/cddis.2017.342 (PMC5550883; doi:10.1038/cddis.2017.342)

S1 Fig. Sorcin binds chemotherapeutic drugs *in vitro* directly and with high affinity.

(A) SPR OneStep Experiment: Sorcin binds vinblastine, paclitaxel and doxorubicin directly and with high affinity; (B) SPR FastStep experiment: Sorcin binds cisplatin with high affinity.

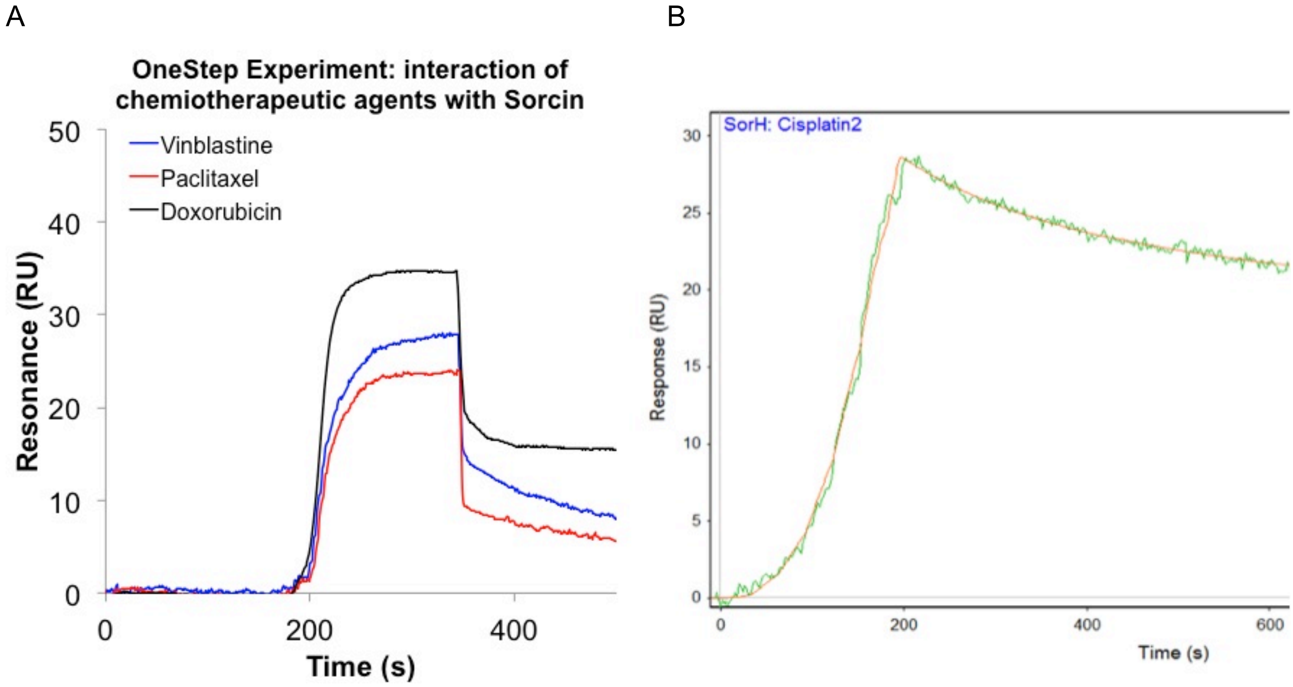

S2 Fig. Sorcin binds doxorubicin with high affinity *in vitro*. Doxorubicin binding to Sorcin monitored by (A) SPR titration experiments in the presence of 500 μM EDTA and (B, C) fluorescence titration experiments in the presence of 1 mM (B) and 5 mM $MgCl_2$. Each protein was incubated for 3 minutes at 25°C in the presence of increasing concentration of ligand. Sorcin contains two binding sites for doxorubicin, with affinities in the nanomolar and low micromolar range.

Sorcin-Doxorubicin	SPR		Fluorescence	
	Site 1 (K_{D1})	Site 2 (K_{D2})	Site 1 (K_{D1})	Site 2 (K_{D2})
EDTA	22 nM	2 μM	0.9 ± 0.5 nM	511 ± 140 nM
+ 500 μM $CaCl_2$	10 nM	1 μM		
+ 1 mM $MgCl_2$			1.2 ± 2.7 nM	360 ± 950 nM
+ 5 mM $MgCl_2$			0.9 ± 1.2 nM	318 ± 280 nM

A

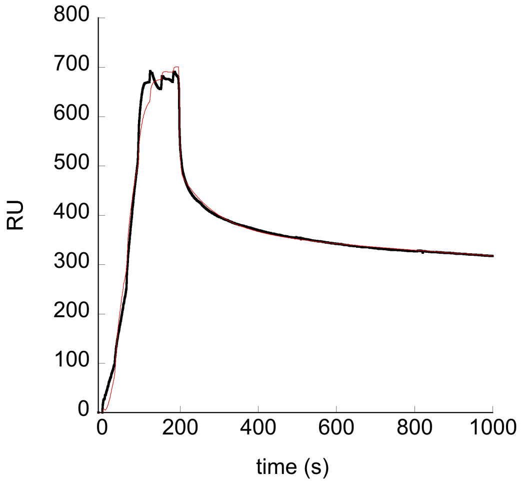

B

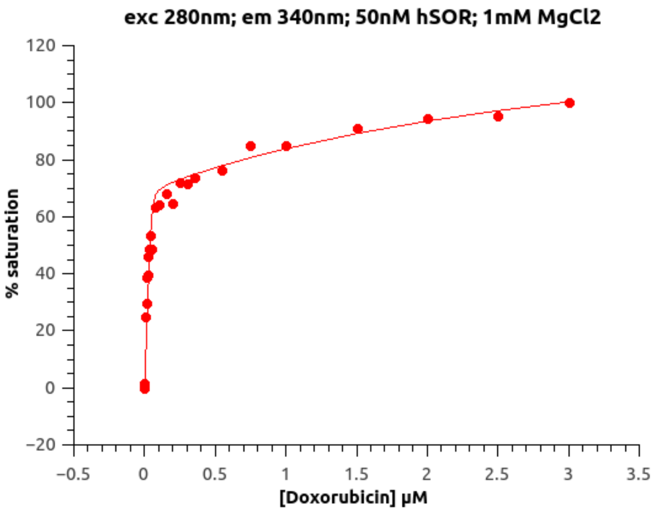

C

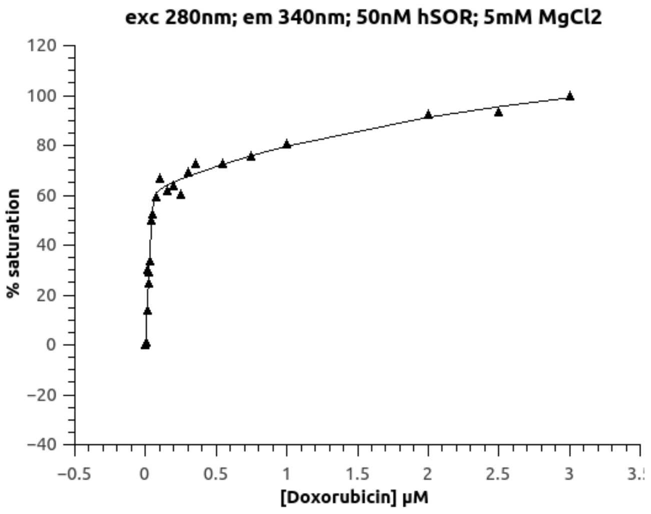

S3 Fig. Emission spectra at 100K (λ_{ex} = 473 nm) of Sorcin crystals containing different amounts of doxorubicin.

Changes in peaks intensity and a 25 nm red shift are likely due to doxorubicin stacking to aromatic residues or dimerization, once bound to Sorcin.

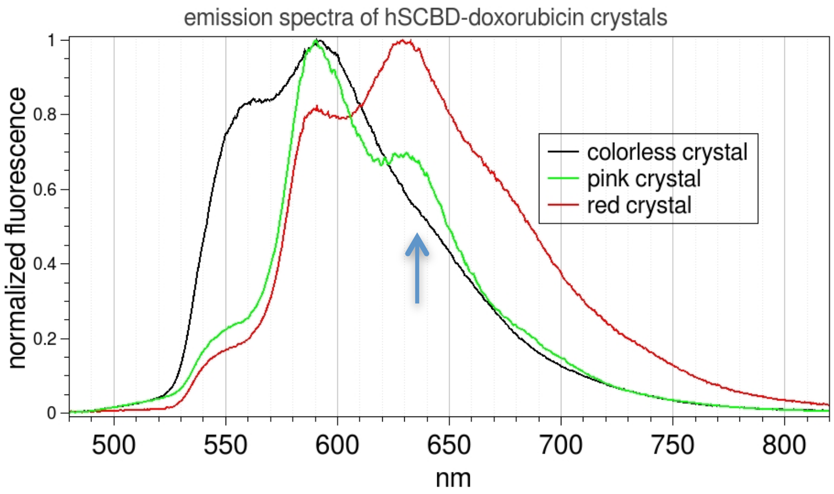

S4 Fig. F_o-F_C omit maps of doxorubicin binding sites.

(A) F_o-F_C omit map contoured at 3σ (blue), calculated in the absence of doxorubicin, showing doxorubicin binding to EF5; the helices surrounding doxorubicin are indicated. (B) blow up of the F_o-F_C omit map contoured at 3σ, showing the peak of density at pocket 2, at the interface between two dimers of the asymmetric unit.

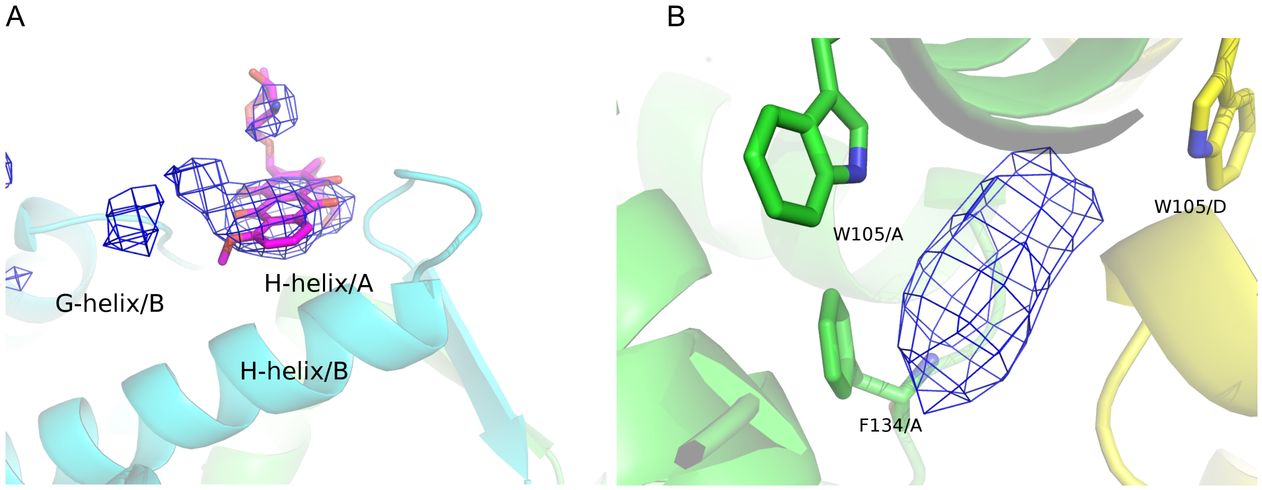

S5 Fig. Sorcin silencing in H1299 cells.

(A) Experiment showing efficiency of Sorcin silencing in control experiments (+Lipofectamine; +scrambled siRNA; + siRNA 3'UTR) vs. silencing using CD53 siRNA. (a and b represent duplicate experiments) (B) Silencing level using CD53 siRNA= -94±3%. Controls vs. 24 and 48 hours treatment with 0.6 μM doxorubicin.

A

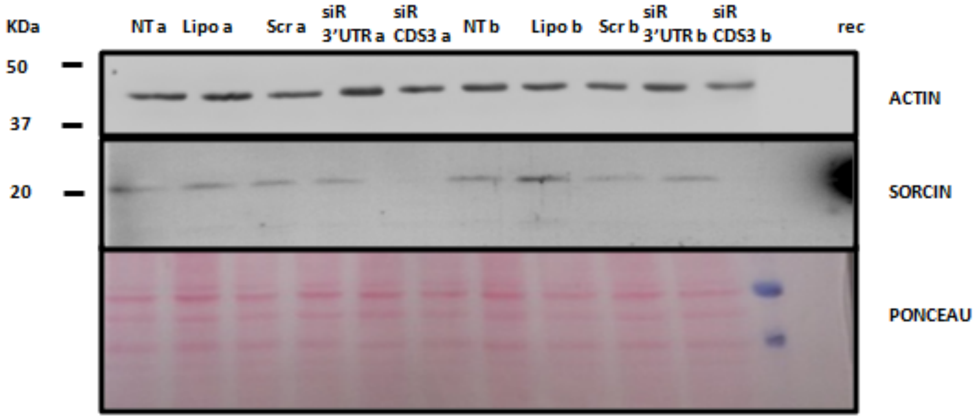

B

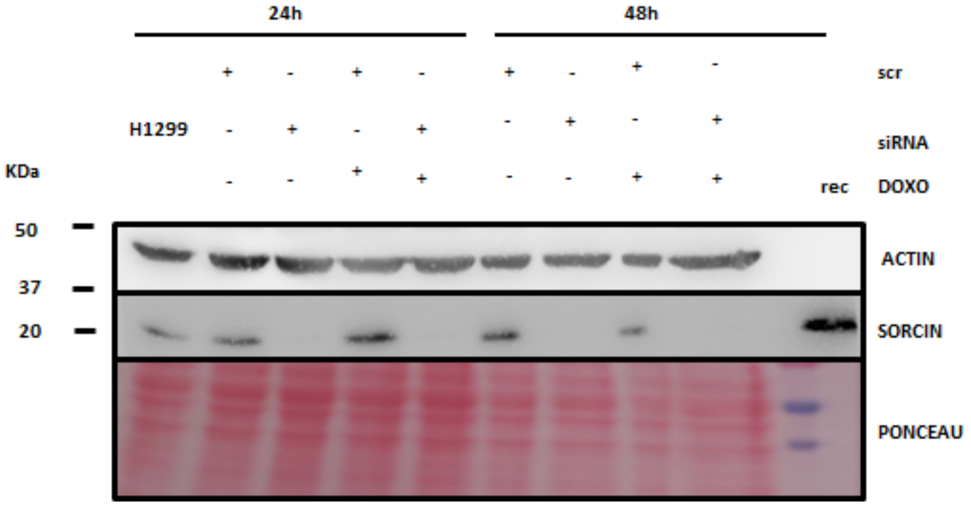

S6 Fig. Experiments of Sytox Blue incorporation.
Cell death percentage upon H1299 cells transfection with scrambled siRNA or Sorcin siRNA in control and 0.6 μM 48 hours doxorubicin-treated cells.

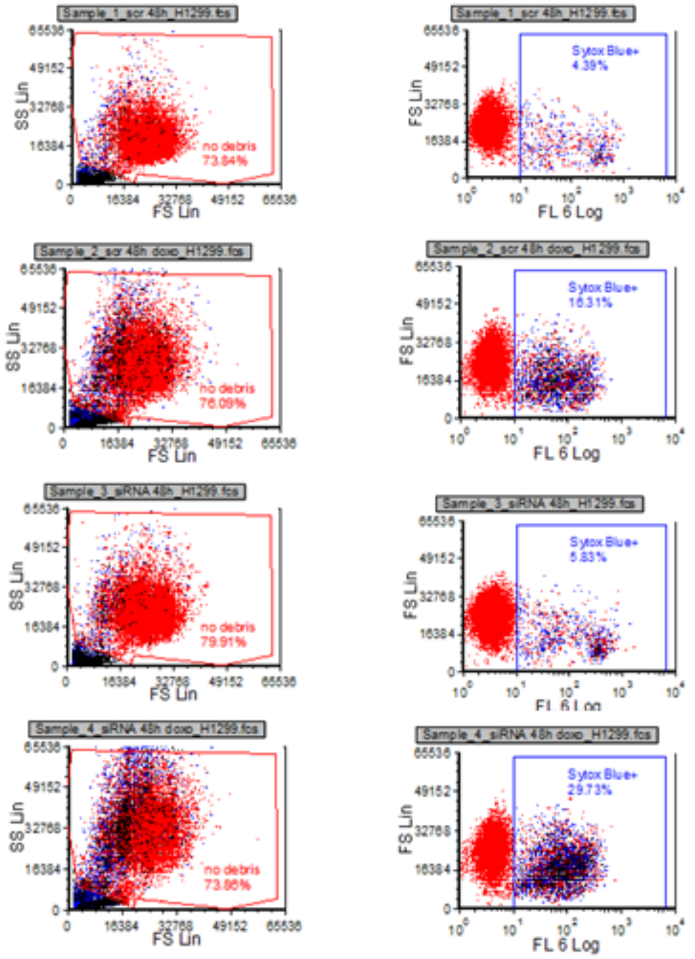

Sample	% SYTOX
scr 48h	4.39
siRNA 48h	5.83
scr doxo 48h	16.31
siRNA doxo 48h	29.73

S7 Fig. Cytofluorimetry experiments showing doxorubicin accumulation.
Doxorubicin accumulation in H1299 cells upon Sorcin silencing (top) with respect to scrambled RNA (below), after 3 hour- (left) and 5-hour treatment (right).

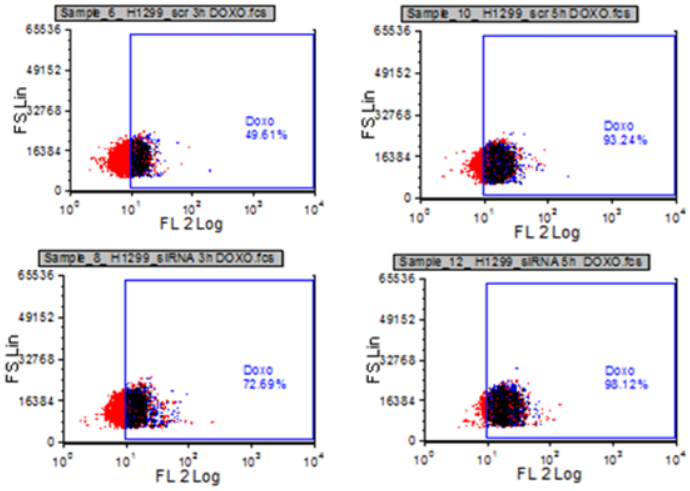

S8 Fig. PARP cleavage experiment upon 48 hours 0.6 μM doxorubicin treatment. Western blot analysis showing that upon Sorcin silencing, there is a negligible cleavage of PARP, compared to the combination of Sorcin-directed-silencing and doxorubicin treatment (PARP FL: PARP full length; PARP CL: PARP cleaved).

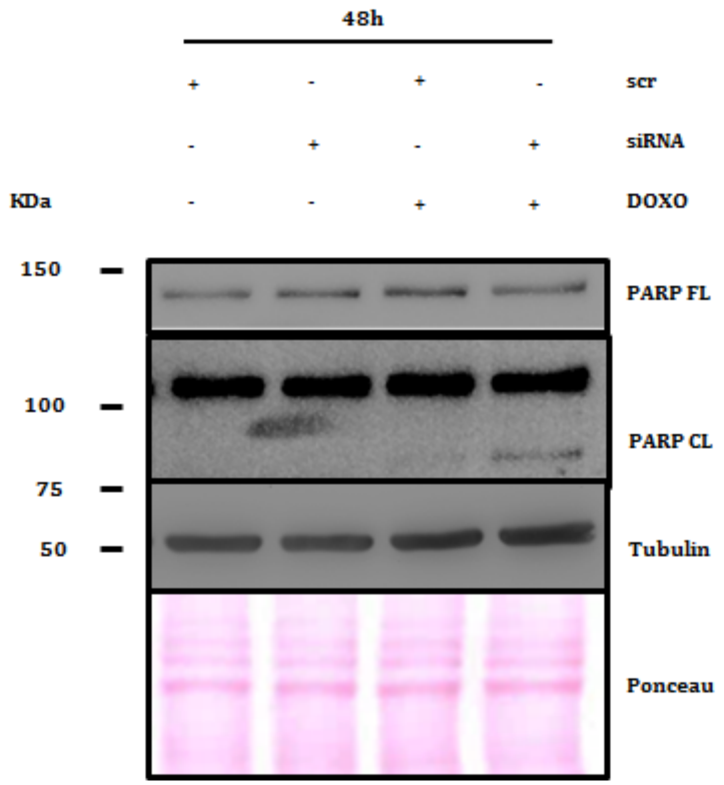

S9 Fig. Sorcin silencing decreases MDR1 expression in H1299 cells.

The experiment shows the incorporation of rhodamine123, in a time-course experiment performed at 37°C, (A) 30’, (B) 1h, (C) 2h after incubation with the dye. Black and blue curves represent the non-treated cells (in the presence of scrambled siRNA and upon Sorcin silencing, respectively), while red and purple curves represent the cells treated with rhodamine123 (in the presence of scrambled siRNA and upon Sorcin silencing, respectively). The experiment shows that rhodamine123 content is increased upon Sorcin silencing, especially after 2h incubation.

A

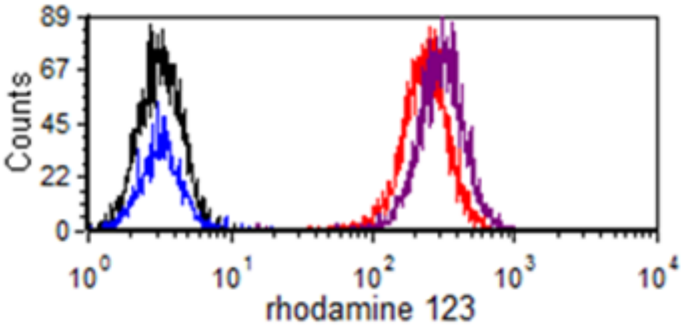

B

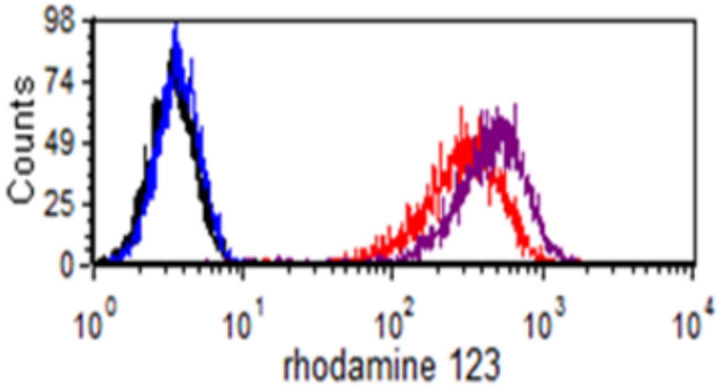

C

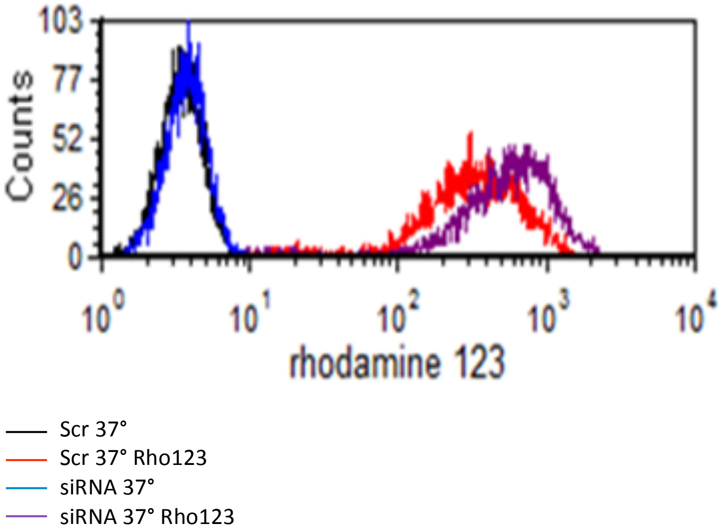

S10 Fig. Sorcin silencing decreases MDR1 expression in H1299 cells.
Western blot experiment showing a decrease in MDR1 protein level upon Sorcin silencing, both 24h and at 48h after treatment with siRNA.

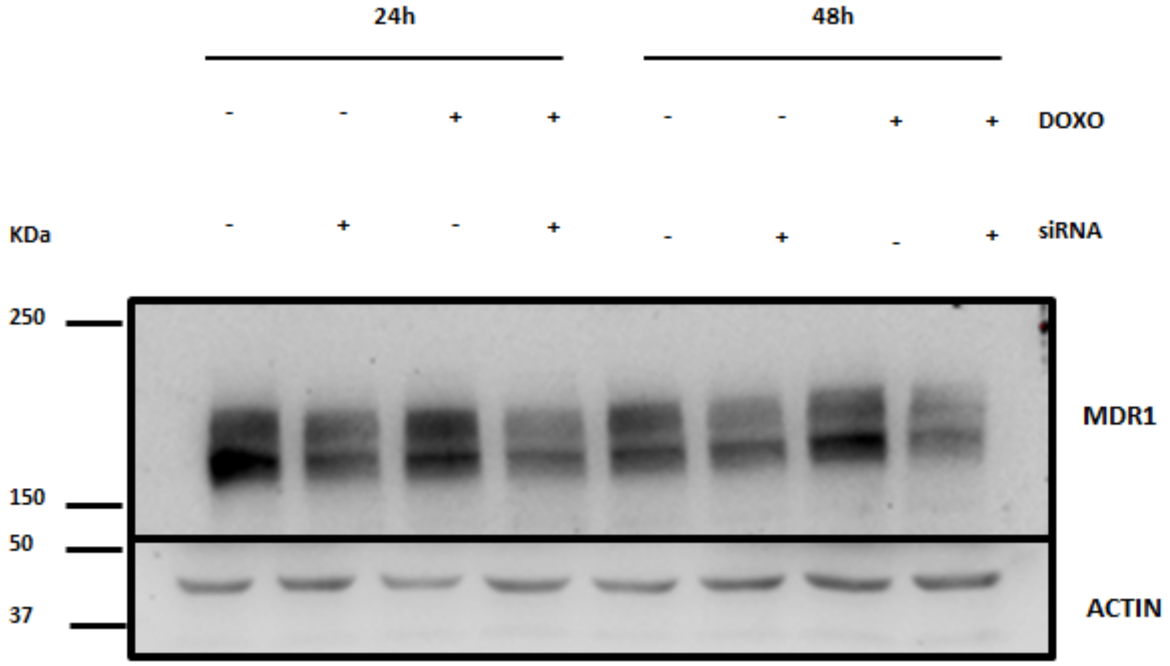

Supplement: Supplementary Material [file cddis2017342x1.pdf]
